# Testing the r^2^SCAN Density Functional for the Thermodynamic Stability of Solids with and without a van der Waals Correction

**DOI:** 10.1021/acsmaterialsau.2c00059

**Published:** 2022-11-09

**Authors:** Manish Kothakonda, Aaron D. Kaplan, Eric B. Isaacs, Christopher J. Bartel, James W. Furness, Jinliang Ning, Chris Wolverton, John P. Perdew, Jianwei Sun

**Affiliations:** † Department of Physics and Engineering Physics, Tulane University, New Orleans, Louisiana70118, United States; ‡ Department of Physics, Temple University, Philadelphia, Pennsylvania19122, United States; § HRL Laboratories, LLC, Malibu, California90265, United States; ∥ Department of Chemical Engineering and Materials Science, University of Minnesota, Minneapolis, Minnesota55455, United States; ⊥ Department of Materials Science and Engineering, Northwestern University, Evanston, Illinois60208, United States

**Keywords:** density functional theory, meta-generalized gradient approximation (meta-GGA), van der Waals interaction, formation enthalpy, decomposistion enthalpy, solid-state materials

## Abstract

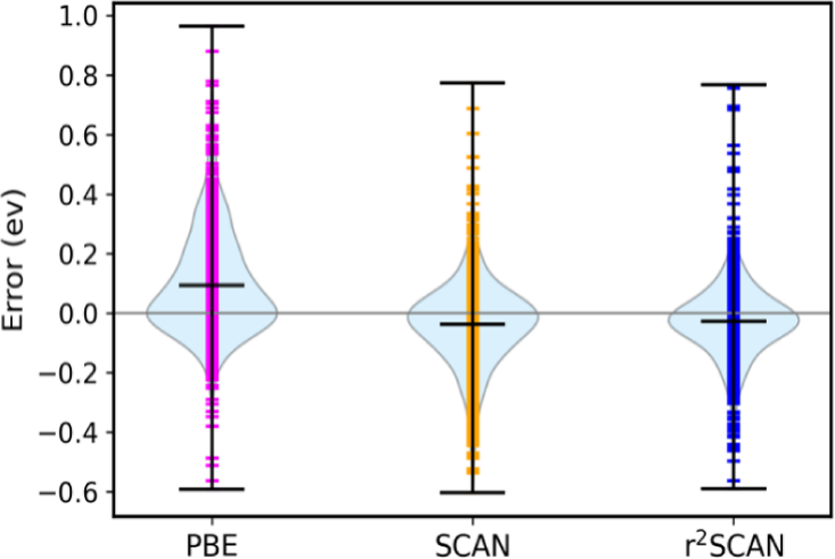

A central aim of materials discovery is an accurate and numerically reliable description of thermodynamic properties, such as the enthalpies of formation and decomposition. The r^2^SCAN revision of the strongly constrained and appropriately normed (SCAN) meta-generalized gradient approximation (meta-GGA) balances numerical stability with high general accuracy. To assess the r^2^SCAN description of solid-state thermodynamics, we evaluate the formation and decomposition enthalpies, equilibrium volumes, and fundamental band gaps of more than 1000 solids using r^2^SCAN, SCAN, and PBE, as well as two dispersion-corrected variants, SCAN+rVV10 and r^2^SCAN+rVV10. We show that r^2^SCAN achieves accuracy comparable to SCAN and often improves upon SCAN’s already excellent accuracy. Although SCAN+rVV10 is often observed to worsen the formation enthalpies of SCAN and makes no substantial correction to SCAN’s cell volume predictions, r^2^SCAN+rVV10 predicts marginally less accurate formation enthalpies than r^2^SCAN, and slightly more accurate cell volumes than r^2^SCAN. The average absolute errors in predicted formation enthalpies are found to decrease by a factor of 1.5 to 2.5 from the GGA level to the meta-GGA level. Smaller decreases in error are observed for decomposition enthalpies. For formation enthalpies r^2^SCAN improves over SCAN for intermetallic systems. For a few classes of systems—transition metals, intermetallics, weakly bound solids, and enthalpies of decomposition into compounds—GGAs are comparable to meta-GGAs. In total, r^2^SCAN and r^2^SCAN+rVV10 can be recommended as stable, general-purpose meta-GGAs for materials discovery.

## Introduction

The backbone of modern *ab initio* simulations of solids is practical Kohn–Sham density functional theory (DFT).^1^ Efficient, first-principles approximations to the generally unknown exchange–correlation energy have made rapid advances in solid-state materials physics possible. Within the Perdew–Schmidt^2^ hierarchy of density functional approximations (DFAs), the generalized gradient approximation (GGA), which depends upon the spin densities and their gradients, and the meta-GGA, which further depends on the local kinetic energy spin densities, stand as the most appealing semilocal DFAs. GGAs, like the Perdew–Burke–Ernzerhof (PBE) GGA,^3^ offer reasonable general accuracy at low computational expense.

Meta-GGAs can be more computationally demanding than GGAs, but offer greater accuracy. The strongly constrained and appropriately normed (SCAN) meta-GGA^4^ simulates complex and “strongly correlated” materials well, with no +*U* correction for cuprates,^5,6^ and some transition-metal oxides^7,8^ or with a +*U* significantly smaller than for GGAs.^9,10^ The +*U* correction is often interpreted^11^ as a simple material-dependent self-interaction correction, and we anticipate the development of improved universal self-interaction corrections to SCAN-like functionals with improved properties, including band gaps^12^ within a generalized Kohn–Sham scheme.

Typically, semilocal density functional approximations (local spin-density approximations, GGAs, and meta-GGAs) underestimate barrier heights of transition states due to the self-interaction errors. For gas-phase chemical reactions, the barrier heights are more realistic with SCAN-like meta-GGAs than with GGAs due to a reduction of self-interaction errors, and are improved further by existing self-interaction corrections.^13^ Barrier heights in condensed phases, including barriers to vacancy mobility, have not yet (to our knowledge) been explored with SCAN-like functionals. SCAN accurately simulates complex materials, such as the “strongly correlated” cuprates^5,6^ and transition-metal monoxides,^8^ but suffers well-known numerical instabilities inherent to its construction.^14,15^

**Figure 1:**
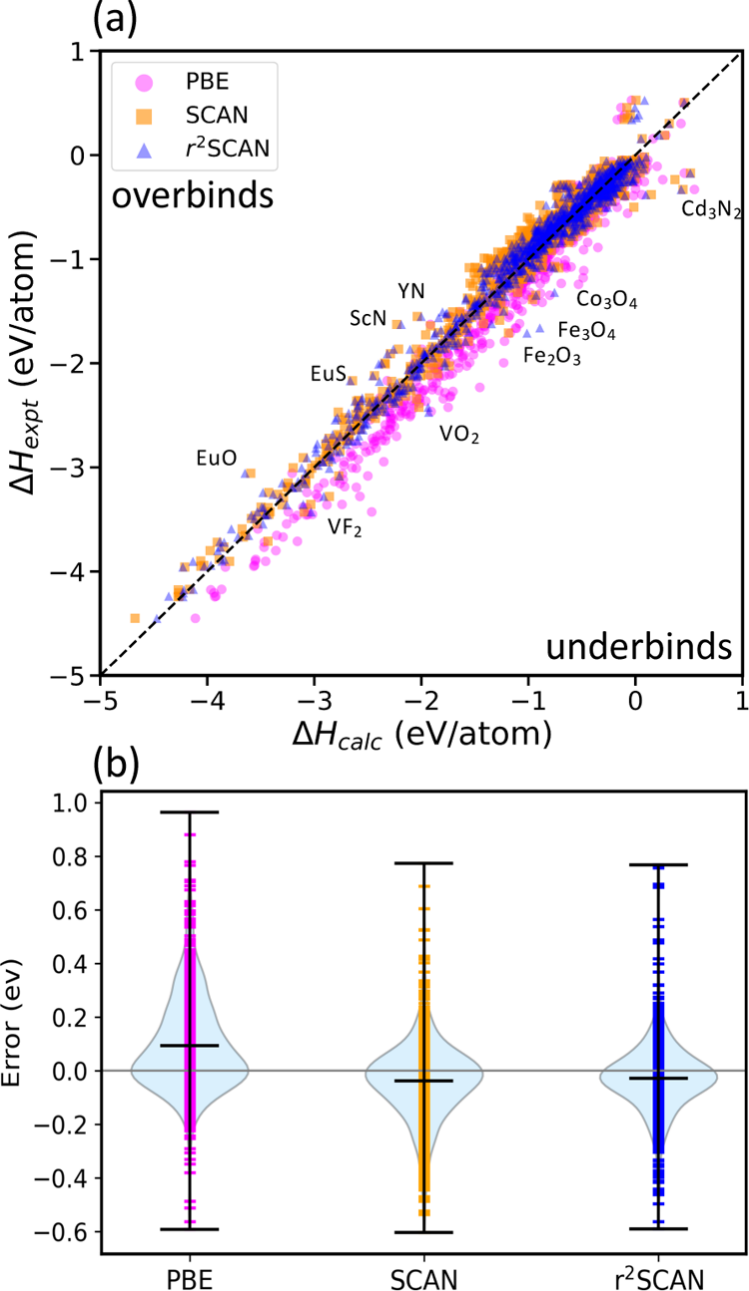
(a) Comparison of calculated and experimental formation energy for the 1015 compounds for PBE, SCAN, and r^2^SCAN. The dashed diagonal line corresponds to the Δ*H*_calc_ = Δ*H*_expt_ line of perfect agreement. (b) Violin plots of the error distributions in the solid set. The r^2^SCAN median error lies closest to zero.

The r^2^SCAN meta-GGA^16^ was constructed as a numerically stable, general-purpose revision of SCAN intended to retain much of its accuracy. r^2^SCAN builds upon the rSCAN meta-GGA of Bartók and Yates^14^ but restores important exact constraints to rSCAN, such as the uniform density limit and coordinate scaling properties.^17^ Tests of r^2^SCAN for molecules^16,18−21^ and for solids^16−18,22,23^ have shown that r^2^SCAN indeed retains or improves upon the high accuracy of SCAN (Figure 1).

The VV10^24^ dispersion correction is a double integral over three-dimensional space of an effective van der Waals interaction between volume elements of the electron density. It is constructed to yield a realistic long-range −*C*_6_/|***r***′ – ***r***|^6^ interaction between well-separated pairs of atoms or molecules. The short-range part of the effective interaction is cut off within a range that can be adapted for use with different semilocal functionals that capture different fractions of the correct intermediate-range interaction. The rVV10^25^ correction emulates the performance of VV10 but (within a plane-wave-based code) avoids the cost of numerical double integration over 3D space.

To promote further progress toward high-throughput meta-GGA calculations for solids, we compare the formation and decomposition enthalpies, unit cell volumes, and electronic structures of more than 1000 solid-state materials calculated using r^2^SCAN, SCAN, and PBE. In addition to SCAN and r^2^SCAN, we present results for their dispersion-corrected^25^ variants: SCAN+rVV10^26^ and r^2^SCAN+rVV10.^27^ The dispersion-corrected r^2^SCAN+D4^20^ describes molecular thermochemistry with exceptional accuracy; however, a broad benchmark of a dispersion-corrected r^2^SCAN in solids has not previously been attempted. As in refs^26^ and (27), we use *b* = 15.7 for SCAN+rVV10 and *b* = 11.95 for r^2^SCAN+rVV10. The *b*-parameter controls the damping of the rVV10 dispersion correction at short range. A larger *b* produces a stronger cutoff. This is needed as semilocal DFAs include a reasonable description of short-range correlation, and meta-GGAs in particular can include an accurate description of intermediate-ranged dispersion interactions through their exchange parts.

## Computational Methods

Calculations of the enthalpies of formation are performed for 934 binary compounds and 81 ternary compounds (see Table S1 in the Supporting Information for chemical formulas). The structures and reference formation enthalpies for these 1015 compounds are taken from the datasets of Isaacs et al.^28^ and Zhang et al.^29^ Reference structures and enthalpies of decomposition for 987 compounds are taken from the dataset of Bartel et al.^30^

All calculations are performed using the Vienna Ab initio Simulation Package (VASP)^31−34^ using the projector augmented wave (PAW) method. A plane-wave energy cutoff of 600 eV is used. Γ-Centered, uniform Monkhorst–Pack *k*-point meshes with *k*-point density of 700 *k*-points per Å^–3^ are generated with pymatgen.^35^ First-order Methfessel–Paxton smearing^36^ of width 0.2 eV is employed for structural relaxations, while total energy calculations use the tetrahedron method with Blöchl corrections.^37^ The algorithm used in our work is the Kosugi algorithm,^38^ which is a special case of the block Davidson iteration scheme. We compare three semilocal exchange–correlation density functional approximations (DFAs): the PBE GGA,^3^ the SCAN meta-GGA,^4^ and the r^2^SCAN meta-GGA.^16^ As no meta-GGA pseudopotentials are available in VASP, we use the “PAW 52” PBE pseudopotentials. In magnetically active systems, the ferromagnetic ordering is considered to be the ground state. For the systems CrB, CoF_2_, CNiO_3_, F_2_Mn, Fe_2_O_3_, Fe_3_O_4_, Fe_4_Ni_2_O_8_, and NiSO_4_ antiferromagnetic orderings are considered. For structure relaxation, the calculations are converged to 10^–6^ eV in the total energy, and 0.01 eV/Å in the atomic forces. For computing formation enthalpies, all calculations are converged to 10^–7^ eV in the total energy, and 0.01 eV/Å in the atomic forces. Molecular reference states are used for H_2_, N_2_, O_2_, F_2_, and Cl_2_, where the isolated molecule is represented by a dimer in a 15 × 15 × 15 Å^3^ box. Experimental standard enthalpies of formation used to determine the error in formation energy are defined at 298 K and 1 atm of pressure.^28^

**Figure 2:**
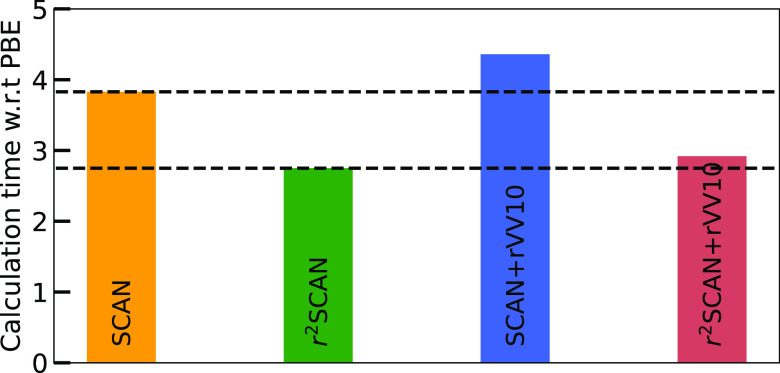
Average calculation time for all functionals considered here relative to that of PBE.

Starting from the PBE geometries, but without using the converged PBE wavefunction, the average calculation times, with respect to those of PBE, on a 35-solid subset of the original 1015-solid set are compared in Figure 2. On average, SCAN (r^2^SCAN) calculations take 3.8 (2.7) times as long as PBE calculations. Starting from the PBE relaxed structures, an average of 36 (28) steps for SCAN (r^2^SCAN) were needed to attain convergence. This agrees well with the similar analyses of refs^28^ and (22). The National Energy Research Scientific Computing Center (NERSC) supercomputing center, and the Extreme Science and Engineering Discovery Environment (XSEDE) high-performance computing resources were used to calculate DFT total energies. The CPU version of VASP-6.2.1 was used in these calculations with 4 nodes and 32 cores.

Reference^28^ found 24 solids, mostly containing transition metals, lanthanides, or transuranics, for which they could not converge their SCAN calculations. These solids are Pu_2_O_3_, PuCl_3_, PuOCl, PuF_3_, PuI_3_, PuOI, PuOF, Ce_2_SO_2_, CeAlO_3_, CeAu, CeCl_3_, CePd, CeSi, UAl_3_, UAl_4_, UFe_2_, UGe_2_, UGe_3_, UI_4_, UN, Sc_2_C, TiFeO_3_, WO_2_Cl_2_, and WCl_4_. While we were able to converge the SCAN calculation of WCl_4_, all other SCAN calculations for this subset failed to converge. r^2^SCAN reliably converged all solids in this set which do not contain Ce, and could converge CeCl_3_. It is not clear why r^2^SCAN did not converge for almost all of the Ce-containing compounds; however, this presents an obvious test of future meta-GGAs.

## Results and Discussion

### Formation Enthalpy

To systematically compare the performance of DFAs on formation enthalpies of solid-state materials, we group databases according to Isaacs et al.^28^ and Zhang et al.^29^ The total set comprises 1015 solids. Figure 1a, which compares experimental and calculated formation enthalpies, shows that PBE systematically underbinds solids, whereas SCAN and r^2^SCAN tend to overbind “weakly bound” solids (|Δ*H*_expt_| ≲ 1 eV/atom). Violin plots of the PBE, SCAN, and r^2^SCAN error distributions are shown in Figure 1b. The PBE error distribution is strongly skewed toward positive errors (predicting too small absolute formation enthalpies), indicating systematic underbinding. SCAN errors are much less systematic but show a tendency to slightly overbind. The r^2^SCAN median error lies closest to zero, and the error distribution is more symmetric than SCAN’s.

While we have performed calculations using the rVV10 counterparts of SCAN and r^2^SCAN, they are not presented in Figure 1 for reasons of clarity. Scatter plots of the errors made by the rVV10-corrected meta-GGAs are given in Figure S1 in the Supporting Information.

**Figure 3:**
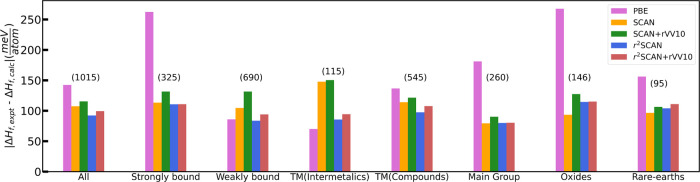
Comparison of mean absolute errors for PBE, SCAN, SCAN+rVV10, r^2^SCAN, and r^2^SCAN+rVV10 with respect to experimental values for formation enthalpies of solids. The 1015 set is partitioned into subsets defined in the text. The numbers in parentheses above each set of bars indicate the number of compounds in that subset.

To better gauge the accuracy of predicted formation enthalpies, Figure 3 presents errors for subsets of the database. “All” is the entire 1015 solid set; “strongly bound” solids have experimental formation enthalpies −4 ≤ Δ*H*_expt_ ≤ −1 eV/atom; “weakly bound” solids have |Δ*H*_expt_| < 1 eV/atom. Transition-metal (TM)-containing compounds are grouped into TM(Intermetallics), which are intermetallics composed only of transition metals, and TM(Compounds), which contain other elements. Main-group solids contain elements from the main group (groups 1, 2, and 13–18 of the periodic table). Oxides are oxygen-containing solids, and rare earths contain at least one rare-earth element (lanthanide series, Sc and Y).

We define a few statistical error metrics that will be used throughout: mean error (ME) or mean deviation (MD)
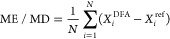
1where *X*_*i*_^DFA^ is a quantity (energy difference, volume, band gap, etc.) computed with a DFA, and *X*_*i*_^ref^ is a reference value. We assume *N* quantities belong to a set. We use “error” to indicate that a reference value is known with very low uncertainty and high accuracy. We use “deviation” when comparing quantities between different approximate methods. The mean absolute error (MAE) or deviation (MAD) is
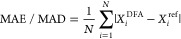
2When analyzed in conjunction with the MAE/MAD, the ME/MD is useful for determining the degree to which a DFA makes systematic errors. If |ME| = MAE, a DFA makes wholly systematic errors. If |ME| ≈ 0, a DFA makes essentially random errors. The root-mean-squared error (RMSE) or deviation (RMSD)
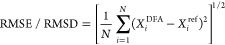
3is a metric comparable to the MAE/MAD. The RMSE/RMSD is simply the square root of the variance. The MAE/MAD is more frequently used than the RMSE/RMSD; however, both carry important information.

For the entire set, the 92 meV/atom MAE of r^2^SCAN is the lowest of all considered DFAs, including r^2^SCAN+rVV10 (99 meV/atom MAE). SCAN has a modestly higher 107 meV/atom MAE for the entire set. For strongly bound compounds, r^2^SCAN, r^2^SCAN+rVV10, and SCAN have nearly identical ∼111 meV/atom MAEs. r^2^SCAN and PBE predict the most accurate formation enthalpies for weakly bound solids with 84 and 86 meV/atom MAEs, respectively. SCAN and SCAN+rVV10 find larger errors for these solids with 105 and 132 meV/atom MAEs, respectively. Consistent with refs^28^ and (22), PBE predicts the most accurate formation enthalpies of intermetallics (70 meV/atom MAE), with SCAN and SCAN+rVV10 making substantially larger MAEs, 148 and 150 meV/atom, respectively. r^2^SCAN and r^2^SCAN+rVV10 predict intermetallic formation enthalpies with accuracy much closer to PBE: their MAEs are 86 and 94 meV/atom, respectively.

For transition-metal-containing compounds, r^2^SCAN has the lowest MAE at 97 meV/atom, followed by r^2^SCAN+rVV10, SCAN, SCAN+rVV10, and PBE, with 108, 114, 122, and 137 meV/atom MAEs, respectively. For main-group compounds, r^2^SCAN, SCAN, and r^2^SCAN+rVV10 have nearly identical ∼80 meV/atom MAEs. SCAN is the most accurate DFA for the oxides (94 meV/atom), with r^2^SCAN and r^2^SCAN+rVV10 following closely behind (114 and 115 meV/atom MAEs, respectively). Finally, all meta-GGAs are comparably accurate for the rare-earth-containing compounds, with SCAN making the smallest MAE, 97 meV/atom.

SCAN+rVV10 often predicts markedly less accurate formation enthalpies than SCAN, as is the case for the strongly bound, weakly bound, and oxide compounds of Figure 3. The increase in errors made by r^2^SCAN+rVV10 over r^2^SCAN is generally less pronounced. SCAN already includes a large fraction of intermediate-range dispersion interactions in its exchange functional, indicated by the large *b*-damping parameter. Thus, SCAN+rVV10 often further overbinds solids that SCAN overbinds. r^2^SCAN includes a less comprehensive description of intermediate-range dispersion interactions than SCAN (indicated by the smaller *b* value, or less severe damping). Thus, even in cases where r^2^SCAN overbinds, r^2^SCAN+rVV10 further overbinds, but to a less pronounced extent.

### Volumes

To assess the accuracy of the predicted crystal structures, we compare the computed relaxed volume per atom to experimental values. Table 1 presents errors in the equilibrium volumes predicted by PBE, SCAN, r^2^SCAN, and their rVV10 counterparts. While PBE overestimates volumes by 0.77 Å^3^/atom on average, SCAN underestimates equilibrium volumes by 0.11 Å^3^/atom on average, and r^2^SCAN overestimates them by 0.24 Å^3^/atom. The MAE in equilibrium volumes for r^2^SCAN and SCAN are 0.59 and 0.58 Å^3^/atom, respectively; thus, r^2^SCAN retains the good general accuracy of SCAN.

**Table 1:** Statistical Errors in Equilibrium Volumes (Å^3^/atom) for a Few Density Functional Approximations (DFAs): PBE, SCAN, SCAN+rVV10, r^2^SCAN, and r^2^SCAN+rVV10^a^

DFA	ME (Å^3^/atom)	MAE (Å^3^/atom)	RMSE (Å^3^/atom)
PBE	0.77	0.98	1.80
SCAN	–0.11	0.58	0.96
SCAN+rVV10	–0.32	0.59	0.95
r^2^SCAN	0.24	0.59	1.04
r^2^SCAN+rVV10	–0.11	0.5	0.88

a r^2^SCAN preserves much of the accuracy of SCAN at better computational efficiency. SCAN+rVV10 performs as accurately as SCAN, with a tendency to predict slightly smaller volumes. r^2^SCAN+rVV10 offers a slight improvement over r^2^SCAN.

The rVV10 van der Waals (vdW) correction does not improve upon the volumes predicted by SCAN. However, r^2^SCAN+rVV10 improves slightly on r^2^SCAN with a 0.5 Å^3^/atom MAE. This is again a reflection of the underlying meta-GGA description of dispersion interactions. rVV10 often produces more meaningful corrections to r^2^SCAN than to SCAN because SCAN includes a more substantial description of intermediate-range dispersion interactions. Thus, rVV10 can often overcorrect SCAN.

Notably, r^2^SCAN and SCAN over- and underestimate the volume of CoI_2_ by 7% (2.2 and −2.1 Å^3^/atom), respectively; r^2^SCAN+rVV10 overestimates its volume by only 1.6% (0.5 Å^3^/atom). The volumes of layered materials tend to be more accurate when a vdW correction is used.^26,27^ Thus, using a vdW correction to r^2^SCAN or SCAN can improve cell volumes without harming the accuracy of predicted formation enthalpies and can be recommended for general materials discovery.

### Magnetism

Next, we explore the magnetic properties of the elemental metals Fe, Co, and Ni using PBE, SCAN, and r^2^SCAN. The predicted and experimental saturation magnetizations are shown in Table 2. In all cases, r^2^SCAN predicts larger magnetic moments than SCAN, which in turn predicts larger magnetic moments than PBE. r^2^SCAN and SCAN overestimate the magnetization of Fe by 24 and 17%, respectively, while PBE underestimates it by only 1.8%. In contrast, SCAN’s magnetization for Co (1.72 μ_B_) is closer to the experimental value (1.75 μ_B_) than that of r^2^SCAN (1.78 μ_B_) and PBE (1.59 μ_B_). These results confirm a known tendency^39^ of r^2^SCAN to overestimate magnetic moments.

**Table 2:** Magnetic Moments of Fe, Co, and Ni Computed Using PBE, SCAN, SCAN+rVV10, r^2^SCAN, and r^2^SCAN+rVV10^a^

DFA	Fe (μ_B_)	Co (μ_B_)	Ni (μ_B_)
PBE	2.18	1.59	0.62
SCAN	2.60	1.72	0.72
SCAN+rVV10	2.66	1.77	0.82
r^2^SCAN	2.76	1.78	0.80
r^2^SCAN +rVV10	2.75	1.78	0.79
experiment	2.22	1.75	0.62

a Experimental values are included for comparison.

While SCAN+rVV10 predicts larger magnetic moments than SCAN, r^2^SCAN+rVV10 predicts nearly the same magnetic moments as r^2^SCAN. The local magnetic moments predicted by PBE, SCAN (+rVV10), and r^2^SCAN (+rVV10) for all magnetic systems with magnetic moment greater than 0.1 μ_B_ are shown in Supporting Information Figure S3. r^2^SCAN and SCAN predict 15 and 12% larger magnetic moments (on average) than PBE, respectively; their rVV10 counterparts show slightly lower average magnetic moments in comparison with the meta-GGAs.

### Band Gaps

Here, we consider SCAN, r^2^SCAN, and their rVV10 counterparts for electronic band gap prediction. It is well known that semilocal DFAs such as PBE underestimate the fundamental band gap.^40^ In a GGA or a meta-GGA (when the latter is implemented in a generalized Kohn–Sham scheme), the fundamental band gap for a given DFA equals the ionization energy minus the electron affinity of the solid for the same DFA. The meta-GGA band gaps tend to be slightly more realistic than those of GGAs because the corresponding total energy diference tends to be slightly more realistic.^12^ Both meta-GGAs and hybrids are orbital-dependent functionals and are typically implemented in a generalized Kohn–Sham (GKS) scheme, in which the effective exchange–correlation potential is a differential or integral operator (as in Hartree–Fock theory), and not just a multiplication operator as in the original Kohn–Sham scheme. For this reason, GKS band structures can capture^8,12,41,42^ some or all of a contribution to the true fundamental gap that is missing in the band structures of functionals which depend solely upon the spin densities and their spatial derivatives, such as LSDA or GGA. While the gap opening in the r^2^SCAN meta-GGA is modest, larger and more realistic gap openings can be found from hybrid functionals and from self-interaction corrections.^43,44^

**Figure 4:**
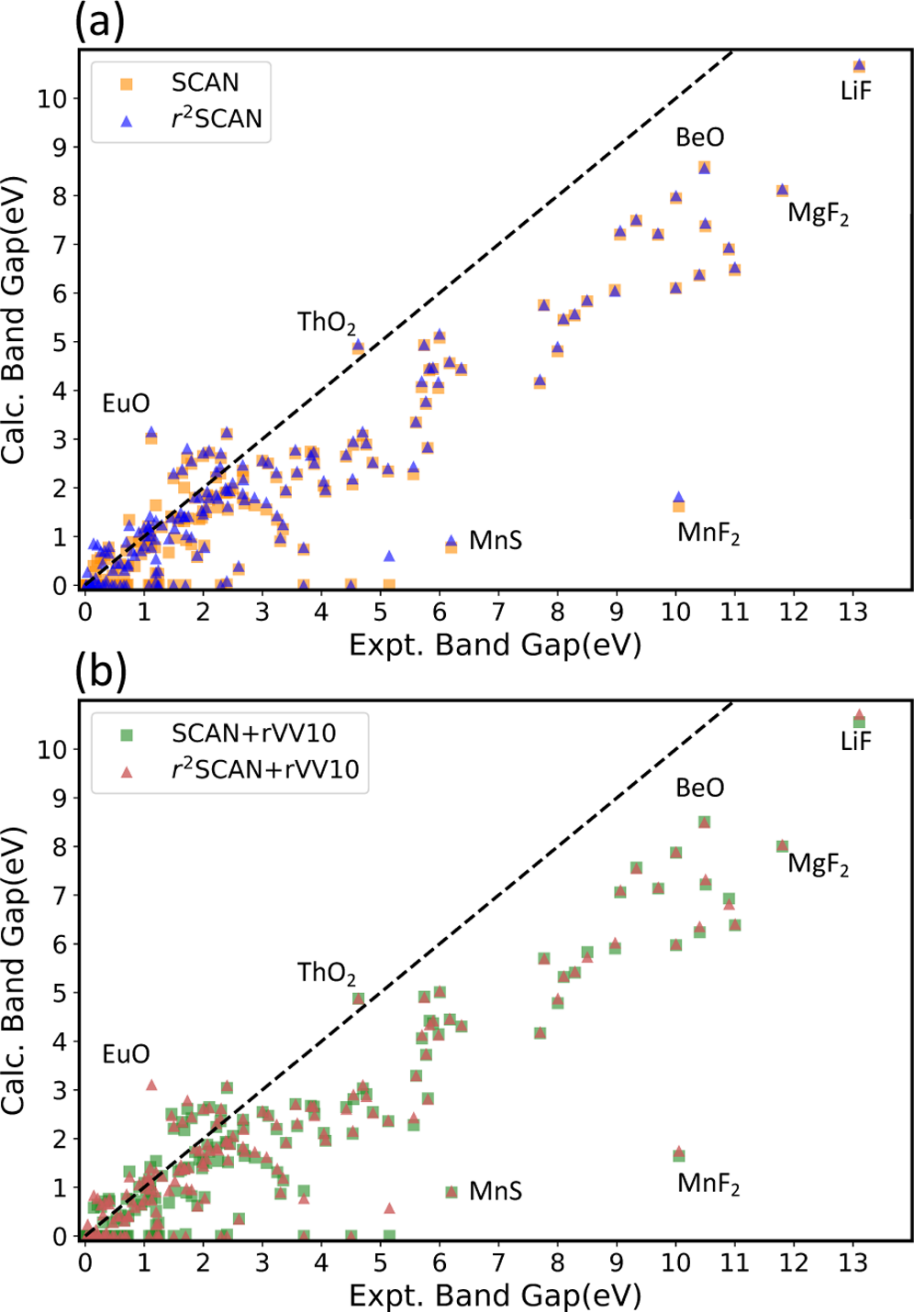
Calculated and experimental electronic band gap. The dashed line corresponds to perfect agreement with the experiment. (a) SCAN and r^2^SCAN; (b) SCAN+rVV10 and r^2^SCAN+rVV10.

Figure 4 compares computed band gaps to experimental values from refs (45) and (46). Nearly all of the points lie below the dashed line of perfect agreement, indicating that r^2^SCAN systematically underestimates the fundamental gap. However, consistent with ref (47), some of the r^2^SCAN gaps are larger than those predicted by SCAN: SCAN predicts WS_2_ to be gapless, whereas r^2^SCAN predicts a 1.41 eV gap, slightly larger than the 1.1 eV experimental gap. Similar trends are seen for the compounds ZnTe, Sb_2_Te_3_, InSe, InSb, InN, InAs, GeTe, FeS_2_, and GaAs.

There are a few systems where r^2^SCAN overestimates the gap more than SCAN underestimates it. For example, SnSe has an experimental band gap of 0.91 eV; SCAN predicts a 0.89 eV gap, whereas r^2^SCAN predicts a much larger 1.06 eV gap. A similar tendency to overestimate the gaps of small-gap insulators has been observed^48^ for the TASK^47^ meta-GGA, which was designed for accurate band gap prediction. The r^2^SCAN band gap tends to be more accurate across all insulators than SCAN’s: r^2^SCAN (SCAN) makes a 1.15 (1.20) eV MAE for this set. The 0.05 eV difference in average errors is largely due to wide-gap compounds such as LiF, MgF_2_, BeO, and MnF_2_. For insulators with an experimental gap less than 5 eV, r^2^SCAN (SCAN) makes a 0.73 (0.77) eV MAE; for insulators with experimental gaps greater than 5 eV, r^2^SCAN (SCAN) makes a 1.36 (1.43) eV MAE.

## Decomposition Enthalpy

Recent studies showed that the signs of decomposition enthalpies are more useful quantities than formation enthalpies for evaluating the stability of compounds.^30,49^ To calculate decomposition enthalpies, we must evaluate the reaction energies of the competing phases of compounds and elements in a composition space.^50−52^ For a given ternary compound ABC, the compound ABC competes with all of the possible elements, binaries, and ternaries in the corresponding A–B–C space. To obtain the decomposition enthalpy of ternary ABC, we compare the energy of ABC with the linear combination of the competing compounds with the same average composition as the ABC compound that minimizes the combined energy of the competing compounds, *E*_A–B–C_. The decomposition enthalpy, Δ*H*_d_, is
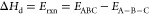
4Δ*H*_d_ > 0 indicates that the ABC compound is unstable with respect to compounds formed from the competing space of A–B–C. Similarly, Δ*H*_d_ < 0 indicates that the ABC compound is stable with respect to its competing phases.

The decomposition reactions that determine Δ*H*_d_ fall into one of three types as defined in ref (30). A type 1 compound is the only known compound in its composition space; the decomposition products are its elemental constituents, and thus Δ*H*_d_ = Δ*H*_f_. For type 2 compounds, the decomposition products are compounds; thus, there are no elemental constituents in the decomposed products. For type 3 compounds, the decomposition products are a combination of compounds and elements.

**Figure 5:**
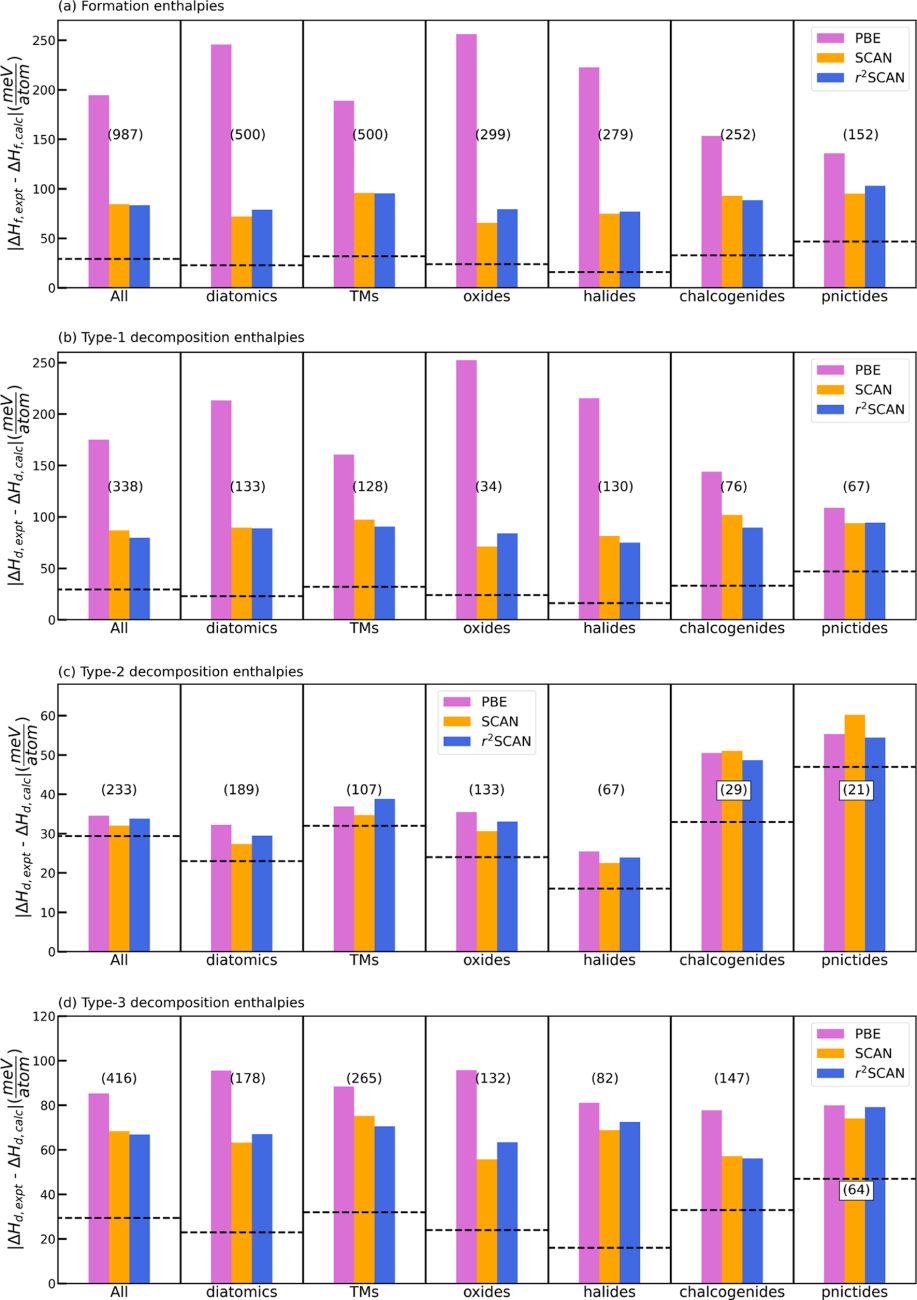
Mean absolute error for PBE, SCAN, and r^2^SCAN taken with respect to experimental values^30,53^ for (a) formation enthalpies, (b) type 1 decomposition enthalpies, (c) type 2 decomposition enthalpies, and (d) type 3 decomposition enthalpies. In addition to the full set (“All”), we consider the diatomic subset, where compounds contain at least one element in the set H, N, O, F, Cl; the TMs subset, compounds that contain at least one element from groups 3–11; oxides, compounds that contain oxygen; halides, compounds that contain at least one element in the set F, Cl, Br, I; chalcogenides, compounds that contain at least one element in the set S, Se, Te; pnictides, compounds that contain at least one element in the set N, P, As, Sb, Bi. The numbers in parentheses above each set of bars indicate the number of compounds in that subset. The dashed horizontal line indicates the approximate uncertainty of Δ*H*_f,expt_ or Δ*H*_d,expt_.

Here, we compare the performance of PBE, SCAN, and r^2^SCAN for the decomposition enthalpies of solid-state materials previously benchmarked by Bartel, et al.^30^ To better elucidate the accuracy of the decomposition enthalpies, Figure 5 presents the errors for subsets of the database. “All” is the entire 987 solid set; “diatomics” contain at least one element in the set H, N, O, F, Cl; “TMs” contain at least one element from groups 3–11; “oxides” contain oxygen; “halides” contain F, Cl, Br, or I; “chalcogenides” contain S, Se, or Te; and “pnictides” contain N, P, As, Sb, or Bi. The total set of 987 solids is partitioned into type 1 (34%), 2 (24%), and 3 (42%) reactions. As shown in Figure 5a, we first analyzed Δ*H*_f_ for all compounds to establish a baseline for subsequent comparison to Δ*H*_d_. The MAE for Δ*H*_f_ is partitioned for various chemical subsets of the dataset in Figure 5a to understand elemental dependence. For this set of 987 compounds, the MAE between the experimentally determined Δ*H*_f_ at 298 K, and calculated Δ*H*_f_ at 0 K, was found to be 194 meV/atom for PBE, 84 meV/atom for SCAN, and 83 meV/atom for r^2^SCAN. PBE shows large systematic errors for a range of diversely bonded systems. SCAN and r^2^SCAN are comparably accurate for all of the partitioned subsets, except for oxides, which are described better by SCAN. The good general accuracy of SCAN is typically attributed to its satisfaction of all 17 known constraints applicable to a semilocal DFA.^54^ r^2^SCAN satisfies one fewer exact constraint than SCAN by recovering a lower-order gradient expansion for exchange than SCAN.^16^ r^2^SCAN’s smoother exchange–correlation energy density could be the reason for its exceptional performance.

To determine the decomposition enthalpies Δ*H*_d_, and thus the thermodynamic stability of compounds, we used Δ*H*_f_ to perform an *N*-dimensional convex hull analysis. We consider only PBE, SCAN, r^2^SCAN, and experimental values. For 338 compounds that decompose as type 1 reactions, Δ*H*_f_ = Δ*H*_d_, the 80 meV/atom MAE of r^2^SCAN is the lowest of all considered DFAs, followed by 87 meV/atom for SCAN, and 175 meV/atom for PBE. As expected, the trend for the type 1 reactions is similar to the overall formation enthalpies shown in Figure 5a. In “real” phase diagrams that are composed only of computed data (e.g., those retrievable in the Materials Project,^55^ OQMD,^56,57^ etc.), there are effectively zero “type 1” compounds because (nearly) every chemical space has at least two calculated compositions.

For the 233 type 2 decomposition reactions, where compounds compete only with other compounds and not elements, r^2^SCAN, SCAN, and PBE are found to perform comparably, with MAEs of ∼35 meV/atom. All DFAs have slightly larger MAEs for the type 2 chalcogenide and pnictide decomposition enthalpies. Specifically for type 2, our results show excellent agreement between experiment and theory for Δ*H*_d_ on a diverse set of materials without requiring an empirical Hubbard-like *U* correction. For the 416 type 3 decomposition reactions, where compounds have elements and compounds that compete energetically, Δ*H*_d_ does not significantly change from SCAN to r^2^SCAN. However, for these compounds, SCAN and r^2^SCAN improve over PBE by ∼20%, and the MAE between r^2^SCAN and experiment (67 meV/atom) falls between those for type 1 (79 meV/atom) and type 2 (34 meV/atom).

## Conclusions

This work has shown that r^2^SCAN^17^ and the dispersion-corrected r^2^SCAN+rVV10^27^ are suitable for general-purpose solid-state materials discovery, in the vein of refs (22, 28, 29). Thus, we highlight conclusions common to previous works and ours, and those that are unique to the work at hand.

Zhang et al.^29^ established that SCAN predicted formation enthalpies of 102 main-group compounds with roughly a factor of 2.5 less average absolute error than PBE. This greater-than-2-fold decrease in MAE is readily confirmed with the larger 260-solid set of main-group compounds presented in Figure 3. Likewise, the formation enthalpies (or type 1 decomposition enthalpies) of Figure 5a,b show a roughly 1.5- to 3-fold decrease in MAE in going from PBE to SCAN or r^2^SCAN.

Figure 3 and Table 2 show that the simple PBE GGA is more accurate than the more sophisticated meta-GGAs for the formation enthalpies and magnetic moments of metals, as observed in earlier works.^28,39,41,58,59^ The reason has been discussed in ref (41). The exact exchange–correlation energy density at a position is proportional to the Coulomb interaction between an electron at that position and the density of the exact exchange–correlation hole which surrounds it. The more short-ranged the hole shape is, the better the functional can be approximated using just the local electron density and its low-order derivatives. Since the long-range part of the exact exchange hole is screened by the long-wavelength dielectric constant of the material, the hole shape is especially short-ranged in metals, where this screening is perfect, and where the local kinetic energy density τ is somewhat too nonlocal. Global hybrid functionals, with the even more nonlocal exact exchange energy density as an ingredient, are even less accurate^59^ for the magnetic moments of metals than meta-GGAs are.

Recall that SCAN recovers more of the intermediate-ranged vdW interaction than does r^2^SCAN. A larger rVV10 *b*-parameter (see also refs^26^ and (27)) more strongly damps the dispersion correction at short range. The SCAN+rVV10 value *b* = 15.7^26^ is much larger than that of r^2^SCAN+rVV10, *b* = 11.95,^27^ indicating that r^2^SCAN needs a more substantial dispersion correction at short to intermediate range than does SCAN. In this sense, rVV10 is a more compatible correction to r^2^SCAN than to SCAN (rVV10 essentially overcorrects SCAN at shorter range). Thus, while Figure 3 often shows marked increases in the MAEs for SCAN+rVV10 over SCAN (see especially the strongly bound, weakly bound, and oxide MAEs), r^2^SCAN+rVV10 essentially does no harm to r^2^SCAN in predicting formation enthalpies. Moreover, r^2^SCAN+rVV10 predicts modestly more accurate cell volumes than r^2^SCAN, as shown in Table 1.

We found that both r^2^SCAN and SCAN tend to underestimate the fundamental band gaps of insulators, as noted previously.^18,22,41^ However, we also found that r^2^SCAN sometimes overestimates the band gaps of narrow-gap insulators. This is consistent with the tendency of the TASK meta-GGA^47^ to overestimate the gaps of narrow-gap insulators.^48^

We confirm the conclusion of ref (22) that r^2^SCAN predicts slightly more accurate formation enthalpies and cell volumes than SCAN. GGAs tend to predict much more accurate formation enthalpies for weakly bound solids, as shown here, and in ref (28) for PBE and SCAN, and in ref (22) for PBEsol,^60^ SCAN, and r^2^SCAN. Likewise, PBE and PBEsol predict much more accurate energetics of transition-metal intermetallics^22,28,41^ than the meta-GGAs, for reasons discussed previously.

The type 2 decomposition enthalpies of Figure 5 show PBE is slightly more accurate than SCAN or r^2^SCAN. However, all DFAs predict type 2 decomposition enthalpies with accuracy close to or below the 30 meV/atom experimental uncertainty. Except for the diatomic and oxide decomposition enthalpies, much smaller decreases in the Type 3 decomposition enthalpies are observed in going from the GGA to meta-GGA level. We have not applied SCAN+rVV10 and r^2^SCAN+rVV10 to the set of solid-state decomposition enthalpies (which differs from the set^28^ presented in Figure 3), for two reasons: (1) this would be computationally cost-prohibitive; and (2) if most solids in the set are strongly bound, a dispersion correction will make insignificant changes to the total energies.

Given the general accuracy and numerical stability of r^2^SCAN^17,18,22^ and r^2^SCAN+rVV10,^27^ it is safe to recommend either for general materials discovery. For metallic systems including intermetallics, the Laplacian of the density is a better ingredient than the orbital kinetic energy density.^41^ When considering layered materials, we recommend r^2^SCAN+rVV10.

## Supporting Information Available

Comparison of the experimentally measured enthalpies of formation with those computed with SCAN and r^2^SCAN, both with and without rVV10 (Figure S1); relative percentage errors in computed formation enthalpies (Figure S2); average magnetic moment computed with PBE, SCAN(+rVV10), and r^2^SCAN(+rVV10) to show the tendency of SCAN-family meta-GGAs to predict larger magnetic moments than PBE (Figure S3); and band gaps computed with SCAN and r^2^SCAN (with and without rVV10) for the insulators considered here (Table S1) (PDF)
