# Supplementary material for: Testing the r2SCAN Density Functional for the Thermodynamic Stability of Solids with and without a van der Waals Correction
Source: ACS Mater Au. 2022 Nov 9;3(2):102–11. doi: 10.1021/acsmaterialsau.2c00059 (PMC9999476; doi:10.1021/acsmaterialsau.2c00059)
Supplement: Supplementary file 1 — mg2c00059_si_002.pdf [file mg2c00059_si_002.pdf]

Testing the r^{2}SCAN density functional for the thermodynamic stability of solids with and without a van der Waals correction

Manish Kothakonda,[†] Aaron D. Kaplan,[‡] Eric B. Isaacs,[¶] Christopher J. Bartel,[§] James W. Furness,[†] Jinliang Ning,[†] Chris Wolverton,[‖] John P. Perdew,[‡] and Jianwei Sun[*,†]

†*Department of Physics and Engineering Physics, Tulane University, New Orleans, Louisiana 70118, United States*

‡*Department of Physics, Temple University, Philadelphia, Pennsylvania 19122, United States*

¶*HRL Laboratories, LLC, Malibu, California 90265, United States*

§ *Department of Chemical Engineering and Materials Science, University of Minnesota, Minneapolis, Minnesota 55455, United States*

‖*Department of Materials Science and Engineering, Northwestern University, Evanston, Illinois 60208, United States*

E-mail: jsun@tulane.edu

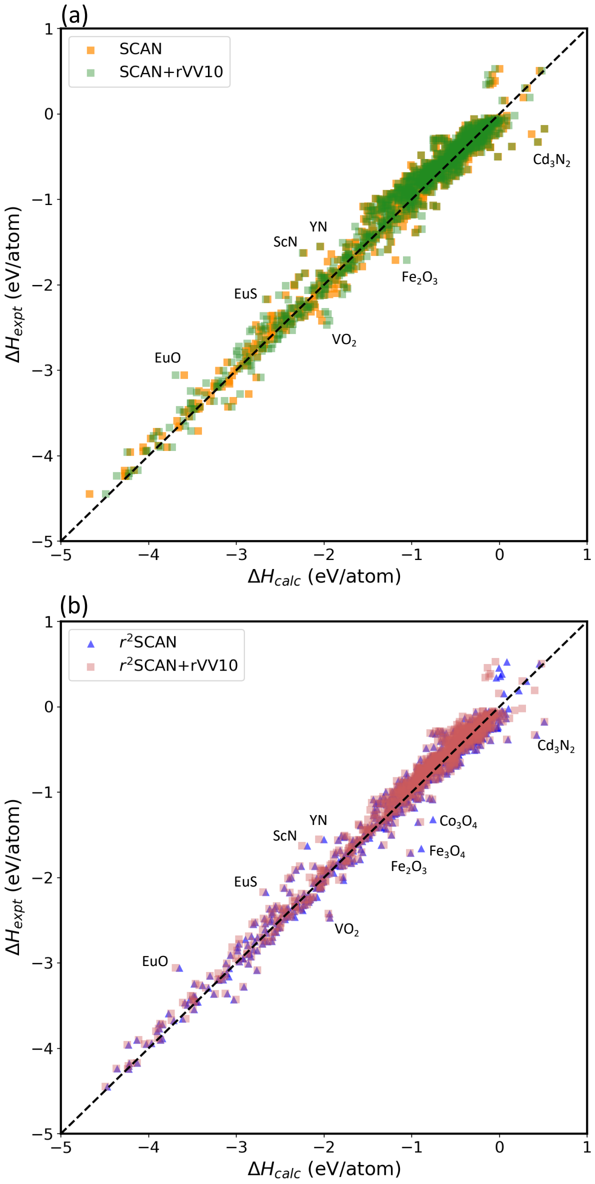

Figure S1: Comparison of calculated and experimental formation enthalpies for the 1015 compounds for (a) SCAN and SCAN+rVV10 (b) r^{2}SCAN and r^{2}SCAN +rVV10. Multiple points for the same compound and functional correspond to different sources of experimental formation enthalpy. The dashed diagonal line corresponds to the $\Delta H_{\mathrm{calc}} = \Delta H_{\mathrm{expt}}$ line of perfect agreement.

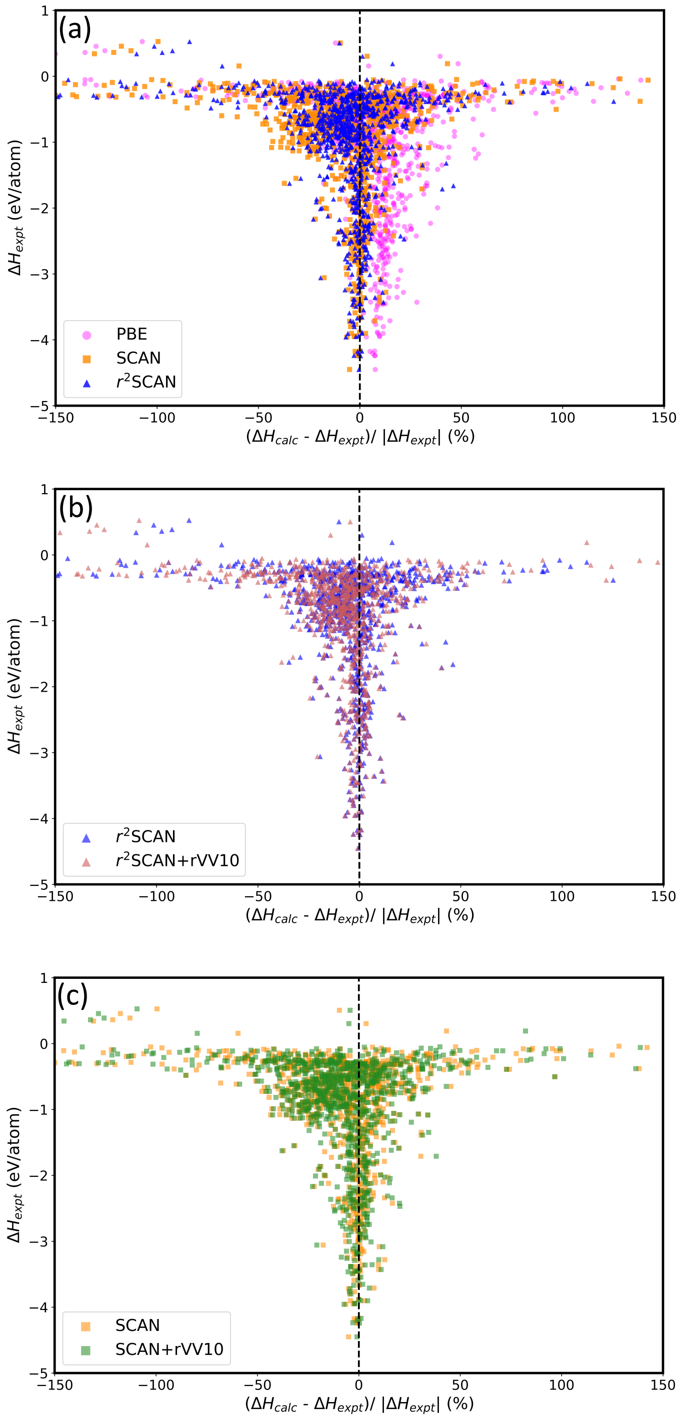

Figure S2: Relative error of the calculated formation enthalpy plotted against the experimental formation enthalpy. The dashed vertical lines correspond to the $\Delta H_{\text{calc}} = \Delta H_{\text{expt}}$ line of perfect agreement. For the relative errors, the range is limited to $\pm 150\%$.

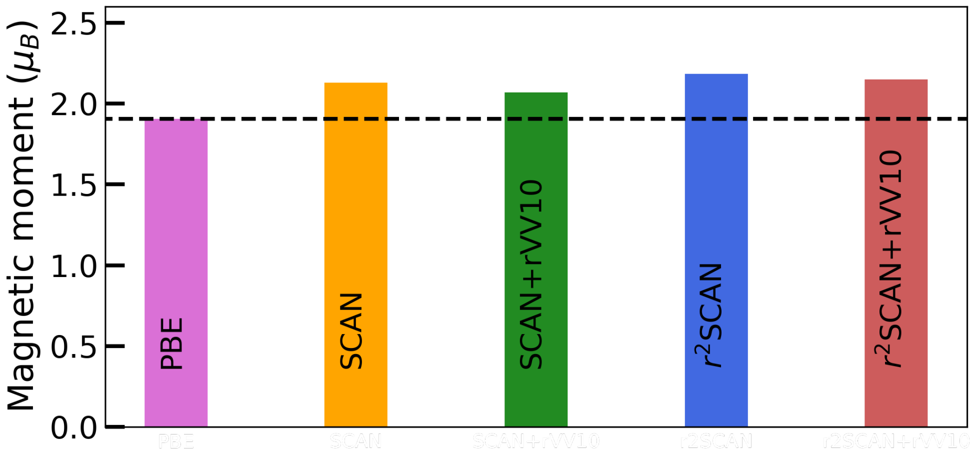

Figure S3: Average Magnetic moment of 157 magnetic compounds, the horizontal line is leveled with PBE magnetic moment to show the difference.

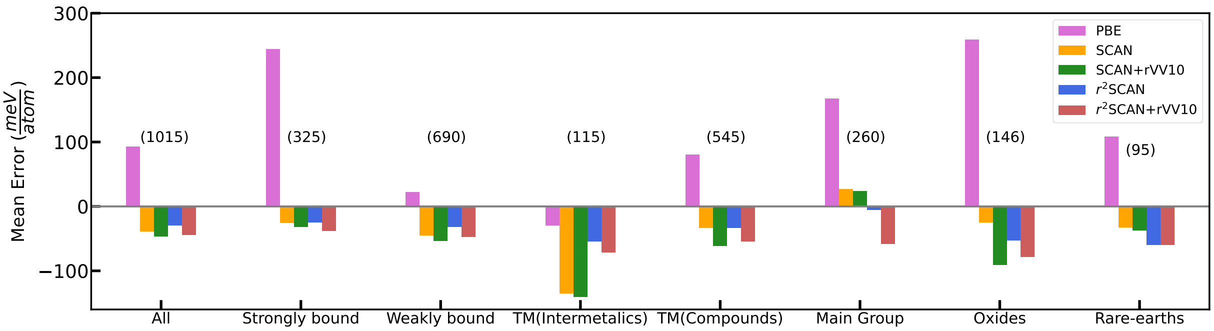

Figure S4: Comparison of Mean Errors for PBE, SCAN, SCAN+rVV10, r^{2}SCAN, and r^{2}SCAN+rVV10 with respect to experimental values for formation enthalpies of solids. The 1015 set is partitioned into subsets defined in the main text. The numbers in parentheses above each set of bars indicate the number of compounds in that subset.

Table S1: Comparison of approximate bandgaps (eV) to experiemnt.

**System**	**Expt**	**SCAN**	**r^2SCAN**	**SCAN+rVV10**	**r^2SCAN+rVV10**
Ag_2O	1.2	0.245	0.261	0.228	0.244
Ag_2S	0.95	0.63	0.713	0.64	0.651
AgCl	3.25	1.346	1.437	1.309	1.383
AgI	2.869	1.641	1.808	1.61	1.727
Al_2O_3	9.7	7.196	7.237	7.137	7.17
Al_2Se_3	3.1	2.502	2.526	2.469	2.489
AlAs	2.1	1.754	1.812	1.728	1.762
AlN	5.74	4.933	4.946	4.915	4.906
AlP	2.45	1.911	1.952	1.888	1.888
AlSb	1.62	1.382	1.497	1.352	1.456
As	1.2	0	0.546	0.514	0.497
AsI_3	2.29	2.297	2.441	2.356	2.42
BaF_2	9.06	7.203	7.288	7.057	7.109
BaO	5.13	2.336	2.41	2.353	2.38
BaS	3.88	2.489	2.525	2.508	2.478
BAs	1.46	1.415	1.404	2.508	2.478
BaTe	3.4	1.915	1.971	1.911	1.926
BeO	10.48	8.591	8.57	8.506	8.499
Bi	0.015	0	0	0	0
Bi_2Se_3	0.21	0.51	0.831	0.637	0.778
Bi_2Te_3	0.145	0.392	0.857	0.579	0.836
BiI_3	1.73	2.615	2.814	2.61	2.787
BN	8	4.804	4.905	4.78	4.882

Continued on next page

Table S1 – continued from previous page

System	Expt	SCAN	r^2SCAN	SCAN+rVV10	r^2SCAN+rVV10
BP	2	1.52	1.467	1.525	1.446
CaB_6	4.5	0.014	0	0	0
CaF_2	10	7.944	8.006	7.87	7.896
CaI_2	5.98	4.051	4.176	4.143	4.136
CaO	7.7	4.148	4.231	4.166	4.193
CaS	5.8	2.816	2.847	2.824	2.816
CaSe	4.87	2.514	2.535	2.541	2.534
CaTe	4.07	1.926	1.979	1.954	1.978
$CdCl_2$	5.7	4.069	4.196	4.06	4.135
CdO	1.2	0	0.075	0	0
CdS	2.4175	1.554	1.631	1.51	1.568
CdSe	1.714	0.925	1.044	0.891	0.981
CdTe	1.517	0.967	1.171	0.925	1.128
CeN	0.7	0	0	0	0
CeO_2	2.68	2.222	2.179	2.224	2.2
CoO	0.47	0.192	0.313	0.287	0.287
CoSi	0.045	0	0	0	0
Cr_2O_3	1.68	2.004	1.418	2.172	1.384
CrO_2	0.23	0	0	0	0
$CrSi_2$	0.35	0	0	0	0
CsCl	8.1	5.447	5.485	5.319	5.342
CsF	10	6.1	6.122	5.972	6.002
CsI	6.37	4.42	4.472	4.302	4.338
Cu_2O	2.023	0.81	0.781	0.799	0.78

Continued on next page

Table S1 – continued from previous page

System	Expt	SCAN	r^2SCAN	SCAN+rVV10	r^2SCAN+rVV10
Cu_2Se	1.23	0	0	0	0
Cu_2Te	1.08	0	0	0	0
CuCl	3.306	0.899	0.98	0.863	0.901
CuI	3.07	1.559	1.707	1.497	1.623
EuO	1.122	3.011	3.164	0	3.111
EuS	1.645	2.309	2.386	2.339	2.348
FeI_2	5.15	0	0.607	0	0.578
FeP_2	0.4	0.769	0.666	0.762	0.666
FeS_2	1.2	1.641	1.333	1.535	1.317
FeSi	0.1	0	0	0	0
$FeTe_2$	0.46	0	0	0	0
Ga_2O_3	4.54	2.884	2.964	2.812	2.906
Ga_2S_3	3.59	2.275	2.337	2.253	2.318
Ga_2Se_3	2.05	1.503	1.605	1.498	1.589
GaAs	1.42	0.669	0.957	0.682	0.937
GaN	3.24	2.221	2.323	2.195	2.291
GaP	2.22	1.824	1.864	1.823	1.809
GaS	2.5	1.985	2.108	2.053	2.057
GaSb	0.725	0.008	0.405	0	0.406
GaSe	1.98	1.388	1.543	1.391	1.517
Ge	0.665	0.138	0.313	0.313	0.313
GeI_2	1.5	2.198	2.306	2.204	2.261
GeO_2	5.56	2.283	2.444	2.278	2.436
GeS	1.58	1.321	1.379	1.322	1.361

Continued on next page

Table S1 – continued from previous page

System	Expt	SCAN	r^2SCAN	SCAN+rVV10	r^2SCAN+rVV10
GeSe	1.1	1.005	1.122	1.048	1.085
$GeSe_2$	2.38	1.897	2	1.934	1.958
GeTe	0.84	0.387	0.619	0.534	0.597
I	1.3	1.194	1.248	1.233	1.233
InAs	0.356	0	0.094	0	0.078
InN	2.4	0.015	0.086	0.028	0
InS	1.86	1.797	1.821	1.721	1.805
InSb	0.17	0	0.044	0	0
InSe	1.187	0.775	0.948	0.778	0.912
K_2S	2.1	2.72	2.774	2.646	2.64
K_2Se	1.8	2.494	2.565	2.416	2.454
K_3Sb	1	0.761	0.817	0.712	0.752
KCl	8.5	5.83	5.864	5.827	5.735
KF	10.9	6.899	6.946	6.928	6.819
KI	6.17	4.559	4.601	4.448	4.469
Li_3Sb	1	1.056	1.185	1.088	1.151
LiCl	9.33	7.483	7.519	7.565	7.558
LiF	13.105	10.643	10.705	10.554	10.722
LiI	6	5.083	5.169	5.01	5.038
Mg_2Ge	0.532	0.359	0.454	0.355	0.415
Mg_2Pb	0.041	0	0.271	0	0.24
Mg_2Si	0.6	0.429	0.456	0.429	0.42
MgF_2	11.8	8.096	8.147	7.998	8.044
MgO	7.77	5.753	5.761	5.702	5.701

Continued on next page

Table S1 – continued from previous page

System	Expt	SCAN	r^2SCAN	SCAN+rVV10	r^2SCAN+rVV10
MgSe	5.6	3.345	3.366	3.287	3.296
MgTe	4.7	3.079	3.162	3.03	3.103
MnF_2	10.05	1.623	1.828	1.639	1.751
MnI_2	4.04	2.048	2.146	2.114	2.109
MnO	3.7	0	0	0	0
MnS	6.2	0.768	0.928	0.894	0.927
MnSe	1.8	0.834	0.992	0.816	0.956
MnTe	1.25	0	0	0	0
MoS_2	1.07	1.207	1.216	1.077	1.216
Na_2S	2.4	3.103	3.157	3.039	3.098
Na_2Se	2	2.656	2.727	2.588	2.623
Na_2Te	2.3	2.612	2.724	2.579	2.625
Na_3Sb	1.1	0.949	1.019	0.91	0.963
NaCl	8.97	6.065	6.047	5.906	6.027
NaF	10.5	7.371	7.442	7.22	7.329
NaI	5.89	4.443	4.479	4.368	4.425
NiO	3.7	0.731	0.779	0.927	0.777
NiS	0.12	0	0	0	0
PbO	1.936	1.359	1.813	1.567	1.757
PbO_2	1.7	0	0	0.037	0
PbS	0.41	0.619	0.802	0.716	0.767
PbSe	0.27	0.555	0.695	0.594	0.644
PdO	1.5	0	0	0	0
PrO_2	0.66	0	0	0	0

Continued on next page

Table S1 – continued from previous page

System	Expt	SCAN	r^2SCAN	SCAN+rVV10	r^2SCAN+rVV10
PtS	0.8	0.888	0.71	0.752	0.71
PtS_2	0.75	1.343	1.237	1.323	1.221
RbCl	8.29	5.538	5.579	5.411	5.436
RbF	10.4	6.36	6.392	6.235	6.358
RbI	5.83	4.415	4.471	4.422	4.348
$ReSi_2$	0.12	0	0	0	0
S	3.82	2.745	2.681	2.669	2.669
Sb	0.1	0	0	0	0
Sb_2Te_3	0.3	0.11	0.449	0.277	0.439
SbI_3	2.22	2.215	2.346	2.237	2.317
ScN	2.6	0.322	0.394	0.354	0.354
Se	1.75	1.372	1.425	1.408	1.407
Si	1.12	0.827	0.787	0.767	0.748
SiO_2	11	6.473	6.538	6.379	6.411
SmS	0.22	0	0	0	0
SnI_2	2.4	1.903	1.964	1.902	1.941
SnO_2	2.7	1.679	1.767	1.642	1.742
SnS	1.08	1.075	1.206	1.167	1.173
SnS_2	2.07	1.855	1.932	1.881	1.889
SnSe	0.91	0.887	1.095	1.006	1.062
$SnSe_2$	1.03	0.933	1.014	0.95	0.992
SnTe	0.18	0.271	0.149	0	0.13
SrO	5.77	3.728	3.785	3.719	3.747
SrS	4.76	2.888	2.93	2.91	2.873

Continued on next page

Table S1 – continued from previous page

System	Expt	SCAN	r^2SCAN	SCAN+rVV10	r^2SCAN+rVV10
SrSe	4.42	2.641	2.693	2.646	2.609
TaN	2.3	0	0	0	0
TaS_2	0.1	0	0	0	0
TbO_2	0.5	0	0	0	0
Te	0.332	0.531	0.71	0.709	0.696
ThO_2	4.625	4.859	4.961	4.876	4.886
Ti_2O_3	0.02	0	0	0	0
TiO_2	3	2.556	2.572	2.541	2.572
TiS_2	1.24	0.23	0.31	0.272	0.271
Tl_2Te_3	0.7	0.706	0.89	0.762	0.86
TlCl	3.56	2.712	2.788	2.707	2.709
TlI	2.67	2.355	2.477	2.386	2.409
TlSe	0.73	0.391	0.446	0.41	0.422
V_2O_3	0.1	0	0	0	0
VO	0.3	0	0	0	0
WS_2	1.1	0	1.423	1.411	1.407
YN	1.9	0.576	0.62	0.621	0.62
ZnI_2	4.53	2.071	2.194	2.102	2.162
ZnO	3.35	1.151	1.252	1.138	1.195
ZnS	3.87	2.704	2.744	2.653	2.672
ZnSe	2.67	1.8	1.893	1.771	1.84
ZnTe	2.25	1.598	1.797	1.534	1.774
ZrC	0.6	0	0	0	0
ZrS_2	1.68	1.314	1.417	1.388	1.383
